# Association Between Blood Lead Levels and Hypertension in a South Indian Population: A Case-Control Study

**DOI:** 10.7759/cureus.22277

**Published:** 2022-02-16

**Authors:** Anirudh Maslekar, Anil Kumar, Vishwanath Krishnamurthy, Ashwin Kulkarni, Megha Reddy

**Affiliations:** 1 Internal Medicine, M. S. Ramaiah Medical College, Bengaluru, IND; 2 Medicine, M. S. Ramaiah Medical College, Bengaluru, IND

**Keywords:** toxicity, systolic blood pressure, lead, hypertension, diastolic blood pressure

## Abstract

Background

Exposure to lead and its accumulation in the body can lead to progressive adverse effects, including increased blood pressure which is associated with the onset of cardiovascular diseases. In this study, we aimed to determine the relationship between blood lead levels and blood pressure. In addition, we compared blood lead levels between hypertensives and normotensives to determine relationships, if any, between lead exposure and high blood pressure.

Methodology

This was a hospital-based, case-control study. In total, 102 individuals (hypertensives = 51, normotensives = 51) were included in this study. Hypertensive patients (defined as systolic blood pressure (SBP) of ≥140 mmHg, diastolic blood pressure (DBP) of ≥90 mmHg, or taking antihypertensive medication for regulating blood pressure) were considered to be study cases and normotensive individuals were considered to be study controls. Blood lead levels were compared between the two groups, and the effects of blood lead levels on SBP and DBP were estimated. The blood lead levels were measured using optical emission spectrometry.

Results

The mean blood lead level among hypertensive individuals (5.5743 ± 1.77 µg/dL) was significantly higher compared to normotensive individuals (4.5029 ± 1.3213 µg/dL, P = 0.001). A positive correlation was detected between blood lead levels and SBP (r = 0.304, P = 0.002). However, no significant correlation was found between blood lead levels and DBP.

Conclusions

Blood lead levels were significantly higher in hypertensive patients compared to normotensive individuals. A significant positive correlation was observed between blood lead levels and SBP.

## Introduction

Lead exposure is a topic of public health concern. Lead is a cumulative toxin, and adults exposed to lead are at an increased risk of developing high blood pressure and renal toxicity [1].

Hypertension is a heterogeneous disorder, and its pathogenesis is not completely understood. Hypertension is greatly influenced by environmental and genetic risk factors [2]. Common risk factors include age, obesity, elevated sodium intake, kidney disease, smoking, alcohol consumption, and a family history of hypertension [3]. Several studies have reported that hypertension is associated with chronic lead exposure [4-8]. However, the mechanism behind lead-induced elevation in blood pressure is complex and poorly investigated.

Lead toxicity is a serious health hazard due to pollution and lack of awareness among the masses. It affects almost every organ system in the body. Of all the organ systems, the nervous system is the most affected, both in children and adults. In adults and children, severe damage to the brain and kidneys resulting in death has been observed to be associated with exposure to lead. High exposure to lead can cause miscarriage, and chronic lead exposure can reduce fertility in men [9].

According to the Prospective Urban Rural Epidemiology (PURE) study, hypertension prevalence in Indian adults aged 35-70 years is 30.7%. It accounts for 4.6% of disability-adjusted life years in the country [10]. The hypotheses behind lead-induced hypertension include impairment of renal function, lower bioavailability of nitric oxide, oxidative stress, hyperactivation of the renin-angiotensin system, and decreased expression of soluble guanylyl cyclase [11,12]. Ideally, there should be no lead detectable in the blood because it has no physiological function. According to guidelines issued by the Centers for Disease Control and Prevention, blood lead levels above 10 μg/dL are considered a cause for concern and should be evaluated [13]. The present study was undertaken to quantify the association between blood lead levels and blood pressure by comparing blood lead levels between hypertensives and normotensives. The secondary objective was to evaluate the effect of blood lead levels on systolic blood pressure (SBP) and diastolic blood pressure (DBP).

## Materials and methods

Study setting

A hospital-based, case-control study was conducted among inpatients and outpatients who attended the Department of General Medicine at a tertiary care hospital between September 2017 and September 2019. The study was approved by the Institutional Ethics Committee (Approval number: ECR/215/INST/KA/2013/RR-16). Informed consent was obtained from all study participants.

Inclusion and exclusion criteria

Hypertensive subjects, defined as SBP of ≥140 mmHg, DBP of ≥90 mmHg, or taking antihypertensive medication for regulation of blood pressure, were considered as study cases. Normotensive, age, sex, and other comorbidities-matched subjects were recruited to the control group. Adults aged >18 years were recruited for participation in the study. Patients with secondary hypertension and pregnant women were excluded from the study.

Sample size calculation

Based on a previous study by Rahman et al., mean blood lead levels in hypertensive individuals were 255 ± 57 µg/L and in normotensive individuals were 139 ± 34 µg/L [11]. In this study, considering an effect size of 0.65, setting α error at 5%, and power at 90%, the sample size was calculated to be 51 in each group.

Study design

A detailed history, including age, gender, occupation, education, alcohol intake, smoking, and physical activity, was obtained from study participants. Physical examination included measurements of height, weight, and waist circumference. All subjects were requested to rest for at least 30 minutes, following which blood pressure was measured using a sphygmomanometer with standard cuff size such that it should encircle 80% or more of the patient’s arm circumference. The subject was in a sitting position with feet flat on the floor, legs uncrossed, and back against the chair with his/her bare arm resting on a standard table or other support to ensure that the midpoint of the upper arm was at the level of the heart. A minimum of three readings were obtained with one-minute intervals between two consecutive readings. The mean of three measurements was considered.

Whole blood samples of approximately 5 mL were obtained by venipuncture. Blood samples were collected in heparinized metal-free tubes and refrigerated until sent to the laboratory. The blood lead levels were measured using optical emission spectrometry at the Ramaiah Advanced Testing Laboratory.

Statistical analysis

Data were analyzed using R 3.6.3 statistical software. The Chi-square test was used to evaluate the association between categorical variables. Mann-Whitney U test was performed to compare continuous variables with non-normal distribution. Because data on blood lead levels, SBP, and DBP were not normally distributed, the correlation was calculated by Spearman’s rank correlation test. Continuous variables were presented as mean ± SD, and categorical variables were presented as absolute numbers and percentages. P-values of ≤0.05 were considered statistically significant.

## Results

The mean age of cases was 40.54 ± 10.28 years, and the mean age of the control group was 40.30 ± 9.76 years. The study sample consisted of 27.45% women and 72.45% men. A large proportion of the sample (approximately 74.5%) did not smoke tobacco, and 66.66% did not consume alcohol. Type II diabetes was observed in 28.43% (29) of the sample. The average body mass index (BMI) among cases was 25.61 kg/m^2^, which was significantly higher than controls (24.02 kg/m^2^, p = 0.002). The average waist circumference among cases was 85 cm, which was higher than controls (81.92 cm, p = 0.04). There was no significant difference between groups regarding fasting blood sugar, serum creatinine, and thyroid-stimulating hormone. There was no significant difference between groups regarding total cholesterol, triglyceride, low-density lipoprotein, and high-density lipoprotein (Table 1).

**Table 1:** Comparison of demographic variables between normotensive and hypertensive individuals. #Continuous variables are presented as mean (SD), and categorical variables are presented as absolute numbers (percentages). *Significant at p-values of ≤0.05. BMI = body mass index; DM = diabetes mellitus; FBS = fasting blood sugar; HDL = high-density lipoprotein; LDL = low-density lipoprotein; TSH = thyroid-stimulating hormone

Variable^#^		Group		P-value
		Case	Control	
Gender	Men	37 (50)	37 (50)	0.99
Smoking	Yes	16 (61.53)	10 (38.46)	0.25
Alcohol	Yes	17 (50)	17 (50)	0.99
Type 2 DM	Present	15 (51.72)	14 (48.27)	0.99
Age (year)		40.54 (10.28)	40.3 (9.76)	0.95
Height (cm)		167.09 (6.84)	167.52 (9.76)	0.53
Weight (kg)		69.9 (8.37)	67.6 (7.33)	0.16
Waist circumference (cm)		85 (9.33)	81.92 (8.31)	0.04*
BMI		25.6 (2.67)	24.02 (2.56)	0.002*
FBS (mg/dL)		110.76 (28.78)	109.05(27.32)	0.75
Serum creatinine (mg/dL)		0.82 (0.17)	0.81 (0.18)	0.88
TSH (µIU/mL)		2.49 (1.103)	2.41 (1.11)	0.7
Blood lead levels (µg/dL)		5.57 (1.77)	4.5 (1.32)	0.005*
Total cholesterol		183.27 (29.2)	183.21 (29.64)	0.99
Triglyceride		167.35 (43.63)	164.19 (34.12)	0.95
LDL		73.96 (22.53)	74.62 (22.04)	0.93
HDL		40.49 (10.87)	47.09 (50.14)	0.98

There was a weak positive correlation between lead levels and SBP (0.26), and the relationship was statistically significant (p < 0.05). There was a negligible positive correlation between lead levels and DBP (0.14), and the correlation was not statistically significant (p > 0.05) (Table 2).

**Table 2:** Spearman’s correlation test to assess the relationship between blood lead levels and SBP and DBP. *Significant at p-values of ≤0.05. DBP = diastolic blood pressure; SBP = systolic blood pressure

	Blood lead levels	
	SBP	DBP
Correlation coefficient*	0.26	0.14
P-value	0.007*	0.14

## Discussion

The present study was conducted among 102 individuals (51 hypertensives and 51 normotensives) to compare blood lead levels between groups. The comparison was performed to assess the association between blood lead levels and blood pressure.

In this study, BMI and waist circumference were significantly higher among cases compared to controls. The average BMI among cases was 25.61 kg/m^2^ and among controls was 24.02 kg/m^2^ (p = 0.003). Similar findings have been reported by previous studies [1,11]. Higher BMI and waist circumference are surrogates of metabolic syndrome and are known risk factors of hypertension and metabolic syndrome. Insulin resistance and central obesity are recognized as the primary factors involved in the pathophysiology of metabolic syndrome. Insulin resistance and the resulting hyperinsulinemia may induce blood pressure elevation by the activation of the sympathetic nervous system and the renin-angiotensin-aldosterone system [14].

The mean blood lead level among cases was 5.57 ± 1.77 µg/dL and among controls was 4.50 ± 1.32 µg/dL. This difference was statistically significant (p = 0.001). These results are similar to the study by Rahman et al. where participants with hypertension had higher blood lead levels (2.04 μg/dL; 95% confidence interval (CI) = 1.94-2.15) than normotensive participants (1.87 μg/dL; 95% CI = 1.77-1.98) (p = 0.03) [11]. We also observed a positive correlation between blood lead levels and SBP, which was statistically significant (r = 0.30, p = 0.002). A weak positive correlation was observed between blood lead levels and DBP, which was not statistically significant (r = 0.144, p = 0.148). In the study by Hara et al. who recruited 12,725 patients, a multivariable analysis was performed [15]. Blood lead doubling was associated with higher (p < 0.001) SBP and DBP (SBP = +0.76 mmHg; 95% CI = 0.38-1.13; DBP = +0.43 mmHg; 95% CI = 0.18-0.68).

The average blood lead level of the sample population in our study was 5.0386 ± 1.63 µg/dL. This is lower compared to the average Indian blood lead level estimated in a meta-analysis by Ericson et al. In their meta-analysis of 31 studies and 5,472 subjects, the average blood lead level was 7.52 µg/dL [16]. A study by Iyer et al., which included a large pan-India cohort of 2,22,668 subjects, observed that the average blood lead level in the Indian population aged 20-40 years was 4.2 µg/dL [10].

Hypertension is a complex disease with multiple causes and risk factors, including family history, age, eating habits, weight, and exercise habits [17]. Lead exposure is one of the major causal factors of hypertension. Given the significant association between blood levels and SBP in the study population, evaluation for lead exposure should be considered while testing for secondary causes of hypertension, especially in patients from geographical areas and occupations with known exposure to lead.

The limitations of our study were that this study was conducted at a tertiary care hospital that caters to a wide array of patients and enrolled cases were from different geographical areas and different occupations. Consequently, the quantum and source of lead exposure were not uniform. Additionally, with this study, it is difficult to prove whether higher lead levels are an exclusive independent risk factor by themselves in hypertensive patients.

Exposure to lead can occur through food and water contamination, and bioaccumulation is a matter of concern [18]. According to the World Health Organization, any concentration of lead in the body is harmful with adverse effects [19]. Hence, it is crucial to check lead exposure and minimize its effect on health.

## Conclusions

Significantly higher blood lead levels were observed in hypertensive patients compared to normotensive individuals. A significant positive correlation was observed between blood lead levels and SBP. However, further studies are needed to confirm whether higher blood lead levels are an independent risk factor for hypertension.
